# The genome sequence of the Violet Copper, *Lycaena helle *(Denis & Schiffermüller, 1775)

**DOI:** 10.12688/f1000research.156485.1

**Published:** 2025-01-10

**Authors:** Clara Pladevall, Roger Caritg, Francisco Cámara, Silvia Carbonell, Jèssica Gómez-Garrido, Fernando Cruz, Marta Gut, Vanesa Arroyo, Benjamin Komac, Roger Vila, Roderic Guigó, Tyler S. Alioto, Manel Niell

**Affiliations:** 1 Andorra Research and Innovation (AR+I), Sant Julià de Lòria, Andorra; 2 Centro Nacional de Análisis Genómico (CNAG), Barcelona, Spain; 3 Centre for Genomic Regulation (CRG), Barcelona, Barcelona, Spain; 4 University of Barcelona (UB), Barcelona, Barcelona, Spain; 5 Institute of Evolutionary Biology (CSIC- Pompeu Fabra University), Barcelona, Spain

**Keywords:** Lycaena helle, Violet Copper, genome sequence, RNA-seq, Lepidoptera

## Abstract

We present a genome assembly from an individual female *Lycaena helle* (the Violet Copper; Arthropoda; Insecta; Lepidoptera; Lycaenidae). The genome sequence is 547.31 megabases in span. The entirety of the genome sequence was assembled into 25 contiguous chromosomal pseudomolecules with no gaps, including the Z and W sex chromosomes. The mitochondrial genome has also been assembled and is 15.5 kilobases in length. Gene annotation of this assembly identified 20,122 protein coding genes.

## Introduction

### Species taxonomy

Eukaryota; Opisthokonta; Metazoa; Eumetazoa; Bilateria; Protostomia; Ecdysozoa; Panarthropoda; Arthropoda; Mandibulata; Pancrustacea; Hexapoda; Insecta; Dicondylia; Pterygota; Neoptera; Endopterygota; Amphiesmenoptera; Lepidoptera; Glossata; Neolepidoptera; Heteroneura; Ditrysia; Obtectomera; Papilionoidea; Lycaenidae; Lycaeninae; *Lycaena*; *Lycaena helle* (Denis & Schiffermüller, 1775) (NCBI: txid2795559).

### Background

The genome of the Violet Copper, *Lycaena helle*, also named by some authors *Helleia helle,* ^1^ was sequenced as part of the Catalan initiative for the Earth Biogenome Project (CBP), ^2^ which aims to produce a detailed catalogue of the genome of eukaryotic species in the territories of the Catalan Linguistic Area. Here we present a chromosomally complete genome sequence for *Lycaena helle*, based on one female specimen from Andorra.

This species has a strong sexual dimorphism. Males are copper orange with an intense violet gloss and with black wing margins and black spots. Females lack this marked iridescence and have smaller forewing black markings.

The life cycle of the Andorran population of *L. helle* begins in June/July when the caterpillar hatches from the egg. In two or three weeks the caterpillar develops and pupates. The pupa hibernates and the imago emerges between May and early June of the following year. The adult butterfly feeds on the nectar of different plant species. During these months, the fertilised females deposit their eggs on the leaf underside of the bistort ( *Bistorta officinalis*), the sole larval host plant.

The species was apparently widely distributed in central Europe during the last glacial period and the early postglacial period. ^3^ During the climatic warming of the postglacial period, it rose to higher altitudes and latitudes, ^3^ being relegated to several isolated populations on mountain valleys in the south of the distribution range. The isolation and the fact that this is a sedentary species ^4^ render *L. helle* susceptible of suffering strong population fluctuations ^5^ and population stochasticity. ^6^ Individuals from different mountain ranges are recovered as different microsatellite clusters and, within these clusters, the degree of differentiation varies considerably. ^3, 7^ The analysis of mitochondrial DNA showed that this species has a relatively high spatial genetic structure (GST= 0.684), although the maximum intraspecific divergence is moderate (0.7%). ^8^

A scenario of genetic erosion has been predicted for *L. helle* in the near future because of climate change. ^7^ The current fragmentation of populations alters habitat connectivity and may cause a decrease in population size with a corresponding loss of diversity through genetic drift, impacting the long-term evolutionary potential of populations. ^9– 12^ Reduced genetic diversity can also undermine a species ability to respond to changing environments, for example, climate change, ^13– 15^ which makes populations more vulnerable to extinction. ^16^

The Andorran population of *L. helle* is estimated to be merely around a few dozen individuals (Ubach & Stefanescu, com. pers.) and is thus subject to extinction in front of any changes that may occur in the environment.

The species is classified as Endangered in the European Red List of Butterflies, ^17^ in the Spanish Red List, ^18^ and as CR (Critically Endangered) in the preliminary Red List of the butterflies of Andorra. ^19^ Its haploid chromosome number is n = 24. ^20^

Here we have generated a high-quality genome assembly and produced long-read RNAseq from wing and leg from the individual employed for genome sequencing.

A Catalan translation of the abstract and a non-specialist summary can be found at https://doi.org/10.5281/zenodo.13378787.

## Methods

### Sample acquisition and nucleic acid extraction

The genome was sequenced from one female *Lycaena helle* specimen (specimen ID ERGA VA AD 01, ToLID ilHelHell1), collected from Bordes d’Envalira, Canillo, Andorra (latitude 42.56, longitude 1.68) on 2021-07-19 by Roger Caritg with a butterfly net, 40 cm diameter. The specimen was processed at the Centro Nacional de Análisis Genómico, and the sample coordinator was Vanesa Arroyo. The specimen was formally identified by Clara Pladevall.

Genomic DNA (gDNA) was extracted from the liquid nitrogen fresh-frozen specimen using the Nanobind tissue kit with the aux insect kit (Circulomics), following the manufacturer’s protocol. Briefly, the specimen was homogenized under cryogenic conditions on dry ice using a mortar and pestle. The pulverized tissue was collected into 1.5 ml tubes and lysed with a Circulomics lysis buffer. After pelleting the debris, the Nanobind disk (Circulomics) was used to bind the gDNA from the fresh supernatant. The high molecular weight (HMW) gDNA eluate was quantified using the Qubit DNA BR Assay kit (Thermo Fisher Scientific), and its purity was assessed using Nanodrop 2000 (Thermo Fisher Scientific) UV/Vis measurements. To determine gDNA integrity, the Femto Pulse (Agilent) Genomic DNA 165 kb kit (Agilent) was employed. The HMW gDNA sample was stored at 4°C.

Total RNA was extracted from liquid nitrogen-fresh-frozen specimens of body parts (wings and legs) using the Quick-RNATM MicroPrep kit (Zymo Research) according to the manufacturer’s protocol. Briefly, the two tissues were homogenized with liquid nitrogen using a mortar and pestle, and then 300 μl of lysis buffer was immediately added to stabilize the samples. Subsequently, 5 U of DNase I was added to remove any potential genomic DNA contamination. The quality of the eluted RNA was first assessed using the Nanodrop One to evaluate its purity and quantified using the Qubit RNA High Sensitivity Kit (Thermo Fisher Scientific). The size distribution and quality of the RNA were determined using the Tapestation High Sensitivity RNA Kit (Agilent). The obtained total RNA was then stored at -80°C until library preparation for sequencing.

### Genome sequencing

The extracted HMW gDNA from *Lycaena helle* was quality controlled, and sequencing libraries were prepared using the 1D Sequencing kit SQK-LSK110 from Oxford Nanopore Technologies (ONT). In brief, 3.0 μg of gDNA underwent end-repair and adenylation using the NEBNext UltraII End Repair/dA-Tailing Module (NEB), followed by ligation of sequencing adapters. The ligation product was purified using 0.4X AMPure XP Beads and eluted in Elution Buffer (ONT).

Sequencing was performed on a PromethION 24 instrument (ONT) using a flow cell R9.4.1 FLO-PRO002 (ONT), with data collection for 100 hours. Quality parameters of the sequencing runs were monitored in real-time using the MinKNOW platform version 21.10.8, and basecalling was performed using Guppy version 5.0.17.

Short-insert paired-end libraries for whole genome sequencing were prepared using the PCR-free protocol and the KAPA HyperPrep kit (Roche). After end-repair and adenylation, Illumina platform-compatible adapters with unique dual indexes and unique molecular identifiers (Integrated DNA Technologies) were ligated. The sequencing libraries were quality controlled on an Agilent 2100 Bioanalyzer using the DNA 7500 assay (Agilent) to assess size and quantified using the Kapa Library Quantification Kit for Illumina platforms (Roche).

For proximity ligation library preparation, the Omni-C kit (Dovetail) was used following the manufacturer’s protocol, with frozen specimens from *Lycaena helle* as the starting material. After reversal of crosslinking, the DNA was purified, followed by the preparation of Illumina-compatible paired-end sequencing libraries. Biotinylated chimeric molecules were isolated using streptavidin beads before PCR enrichment of the library. The library was amplified with 18 PCR cycles using KAPA HiFi HotStart Ready Mix (Roche).

The whole genome sequencing (WGS) and Omni-C libraries were sequenced on Illumina NovaSeq 6000 with a read length of 2×151 bp following the manufacturer’s protocol for dual indexing. Image analysis, base calling, and quality scoring of the run were processed using the manufacturer’s software, Real-Time Analysis (RTA 3.4.4).

### RNA sequencing

After assessing the quality of the extracted total RNA from *Lycaena helle* legs and wings, sequencing libraries were prepared for each tissue using the PCR-cDNA Sequencing kit SQK-PCS111 from Oxford Nanopore Technologies (ONT), employing the TSO method for cDNA library construction. In brief, 200 ng of total RNA was utilized for each sample. The procedure employs a strand-switching technique to isolate complete transcripts, facilitating the detection of splice variants. Utilizing oligonucleotides provided in the kit, we performed complementary strand synthesis and strand switching on full-length poly-A+ RNA. Subsequently, double-stranded cDNA was generated via 14 cycles of PCR amplification, with an 8-minute extension step, using primers containing 5′ tags to facilitate the ligase-free attachment of Rapid Sequencing Adapters. Sequencing was conducted using a minION instrument (ONT) with a flow cell R9.4.1 FLO-MIN106 (ONT), with data collection over 74 hours. Real-time monitoring of sequencing run quality parameters was performed using the MinKNOW platform version 22.03.6, and basecalling was executed using Guppy version 6.0.1-gpu SUP model.

### Genome assembly, curation and evaluation

The genome was assembled with CLAWS v2.1.0. ^21^ In summary, filtlong v0.2.0 ( https://github.com/rrwick/Filtlong) was used to filter low-quality ONT reads out (using -t 50000000000 option to keep only 50Gb of reads).

The assembly was obtained with Nextdenovo v2.4.0 ^22^ and polished with HyPo v1.0.3 ^23^ using both the ONT and Illumina reads. Illumina reads had previously been trimmed with Cutadapt v3.2. ^24^ The resulting polished assembly was then purged using Purge_dups v1.2.5. ^25^ Hi-C reads were mapped and processed following the Dovetail Genomics recommended procedure ( https://omni-c.readthedocs.io/en/latest/fastq_to_bam.html). Upon inspection of the contact map, it was determined that scaffolding was not necessary. Manual curation was performed using the rapid curation tools ( https://gitlab.com/wtsi-grit/rapid-curation ) and PretextView ( https://github.com/wtsi-hpag/PretextView). The mitochondrial genome was assembled as a single circular contig of 15,479 bp using the FOAM pipeline ( https://github.com/cnag-aat/FOAM). In brief, this pipeline mapped unfiltered ONT reads using MINIMAP2 v2.24 to a mitochondrial reference bait ( *Lycaena phlaeas* mitochondrion assembly, HG995187.2) and assembled them with FLYE v2.19 ^26^ options: --meta --scaffold -i 2), then a circular contig of length closer to the mitochondrial reference (16,264 bp) was polished with Illumina reads twice using NEXTPOLISH v1.4.1, ^22^ and finally the circular contig assembly was annotated with MITOS v 2.1.3 ^27^ to determine the position of the trnF gene and orient the mitogenome to start at this gene using the script *orient_mitogenome_v1.pl.* A Hi-C map for the final assembly was produced using bwa mem v0.7.17, ^28^ samtools v1.9 ^29^ and pairtools v.0.3.0 ^30^ in the PreText file format. To assess the assembly metrics, the *k*-mer completeness and QV consensus quality values were calculated in Merqury ^31^ using a meryl k-mer frequency histogram (k=18) computed from the Illumina WGS reads. The genome was analysed within the BlobToolKit environment ^32^ and BUSCO scores ^33, 34^ were calculated. The interactive GC-coverage plot referenced in Figure 3 and interactive contact map referenced in Figure 5 were produced using Nextflow ^35^ DSL2 pipelines “sanger-tol/readmapping” ^36^ and “sanger-tol/genomenote”. ^37^

Table 3 contains a list of relevant software tool versions and sources.

### Genome annotation

Repeats present in the genome assembly were annotated with RepeatMasker v4-1-2 ( http://www.repeatmasker.org) using the custom repeat library available for insecta. Moreover, a new repeat library specific for our assembly was made with RepeatModeler v1.0.11. After excluding those repeats that were part of repetitive protein families (performing a BLAST search against Uniprot) from the resulting library, RepeatMasker was run again with this new library to annotate the specific repeats.

The gene annotation of the species genome assembly was obtained by combining transcript alignments, protein alignments and ab initio gene predictions. RNA from two different tissues (wing and leg) was obtained and sequenced with ONT PCR-cDNAseq. The long reads were aligned to the genome using MINIMAP2 v2.14 ^38^ with the splice option. Transcript models were subsequently generated using Stringtie v2.2.1 ^39^ and merged using TACO v0.7.3. ^40^ High-quality junctions to be used during the annotation process were obtained by running ESPRESSO v1.3.0 ^41^ after mapping with MINIMAP2. Finally, PASA assemblies were produced with PASA v2.5.2. ^42^ The TransDecoder program, which is part of the PASA package, was run on the PASA assemblies to detect coding regions in the transcripts.

Secondly, the proteomes of the Kamehameha, Danaus and “Squinting bush” butterflies and the “swissprot invertebrates” proteins were downloaded from Uniprot in February 2023. Furthermore, we also downloaded the RefSeq proteomes of all butterflies of the superfamily Papilionoidea from the NCBI available in February 2023. These five protein data sets were combined and aligned to the genome using Miniprot 0.6. ^43^ *Ab initio* gene predictions were performed on the repeat-masked assembly with three different programs: GeneID v1.4, ^44^ Augustus v3.5.0 ^45^ and Genemark-ET v4.71 ^46^ with and without incorporating evidence from the RNAseq data. The gene predictors were run with trained parameters for the honeybee, except Genemark, which runs in a self-trained mode. Finally, all the data were combined into consensus CDS models using EvidenceModeler-1.1.1 (EVM). Additionally, UTRs and alternative splicing forms were annotated via two rounds of PASA annotation *updates.* Functional annotation was performed on the annotated proteins with the Pannzer ^47^ online server.

The annotation of non-coding RNAs (ncRNAs) was obtained by running the following steps on the repeat masked version of the genome assembly. First, the program cmsearch v1.1 ^48^ that is part of the Infernal package ^49^ was run against the RFAM database of RNA families v12.0. Additionally, tRNAscan-SE v2.08 ^50^ was run in order to detect the tranfer RNA genes present in the genome assembly. Long-non-coding RNAs (lncRNAs) were identified by filtering the set of PASA-assemblies that had not been included in the annotation of protein-coding genes to retain those longer than 200bp and not covered more than 80% by a small ncRNA. The resulting transcripts were clustered into genes using shared splice sites or significant sequence overlap as criteria for designation as the same gene.

## Results

### Genome assembly

**Figure 1.**
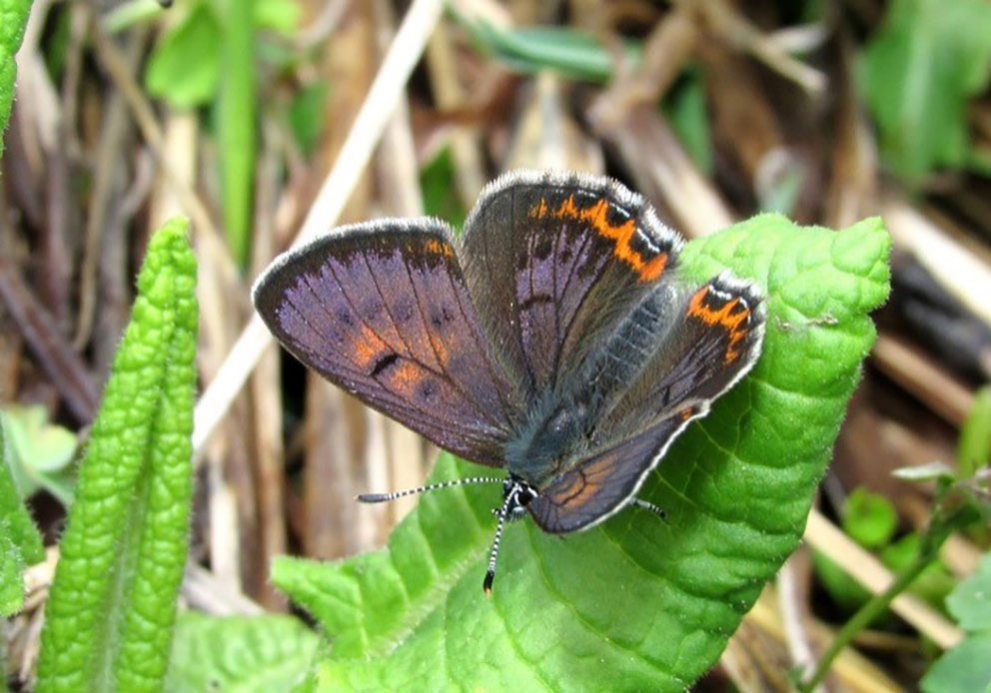
Photograph of the Violet Copper, *Lycaena helle* (photograph by Guy Padfield https://www.guypadfield.com/violetcopper.html).

The genome was sequenced from one female *Lycaena helle* ( Figure 1) collected from Bordes d’Envalira, Canillo, Andorra (latitude 42.56, longitude 1.68). A total of 161-fold coverage in Oxford Nanopore Technologies long reads with an N50 of 41 kb was generated. Pseudohaploid assembly contigs were manually curated using chromosome conformation Hi-C data, correcting only one missing join and one mis-join. The one gap created when correcting the missing join was filled with a 1,541 bp consensus of seven gap-spanning long reads.

**Figure 2.**
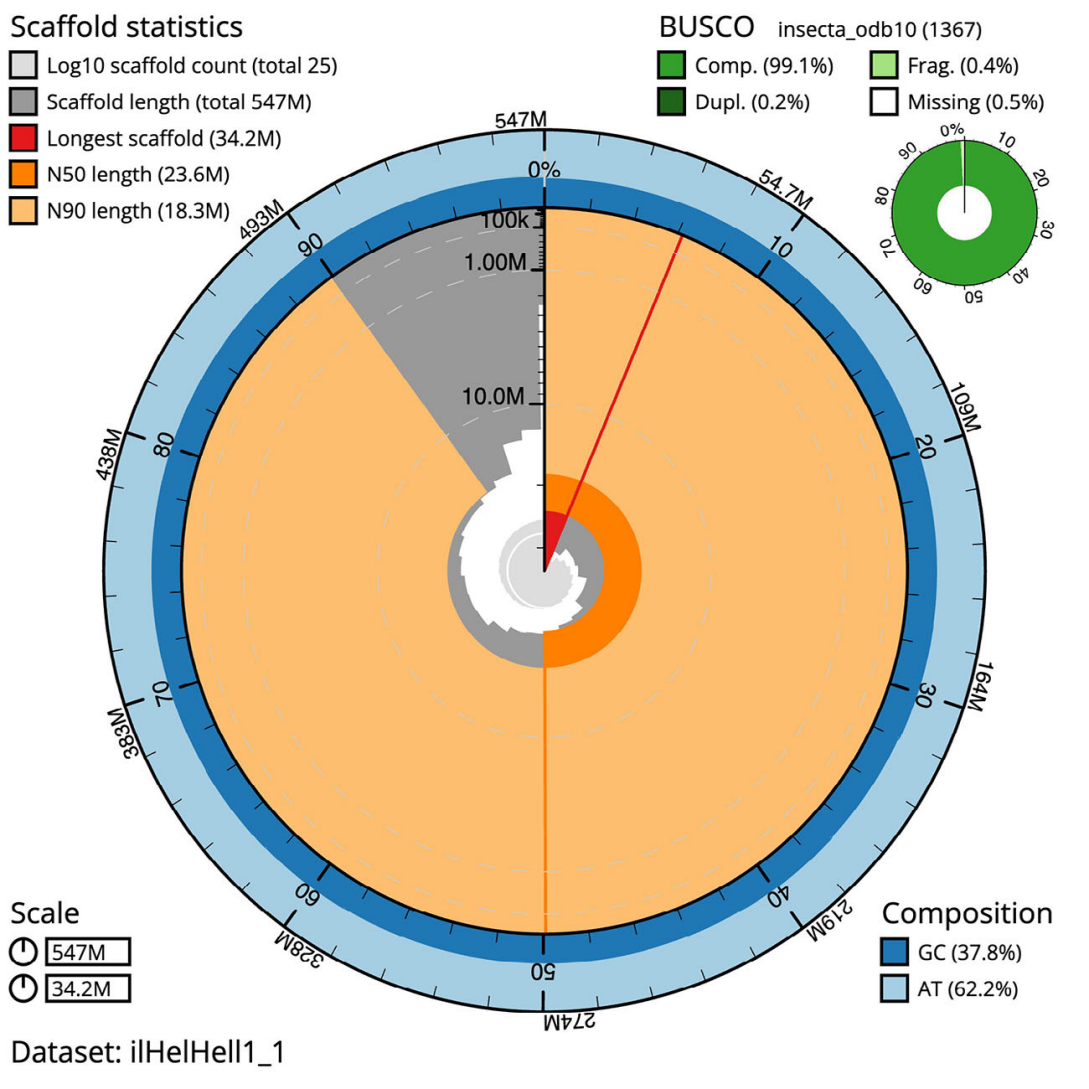
Genome assembly of Lycaena helle, ilHelHell1.1: metrics. The BlobToolKit snail plot shows N50 metrics and BUSCO gene completeness.

**Figure 3.**
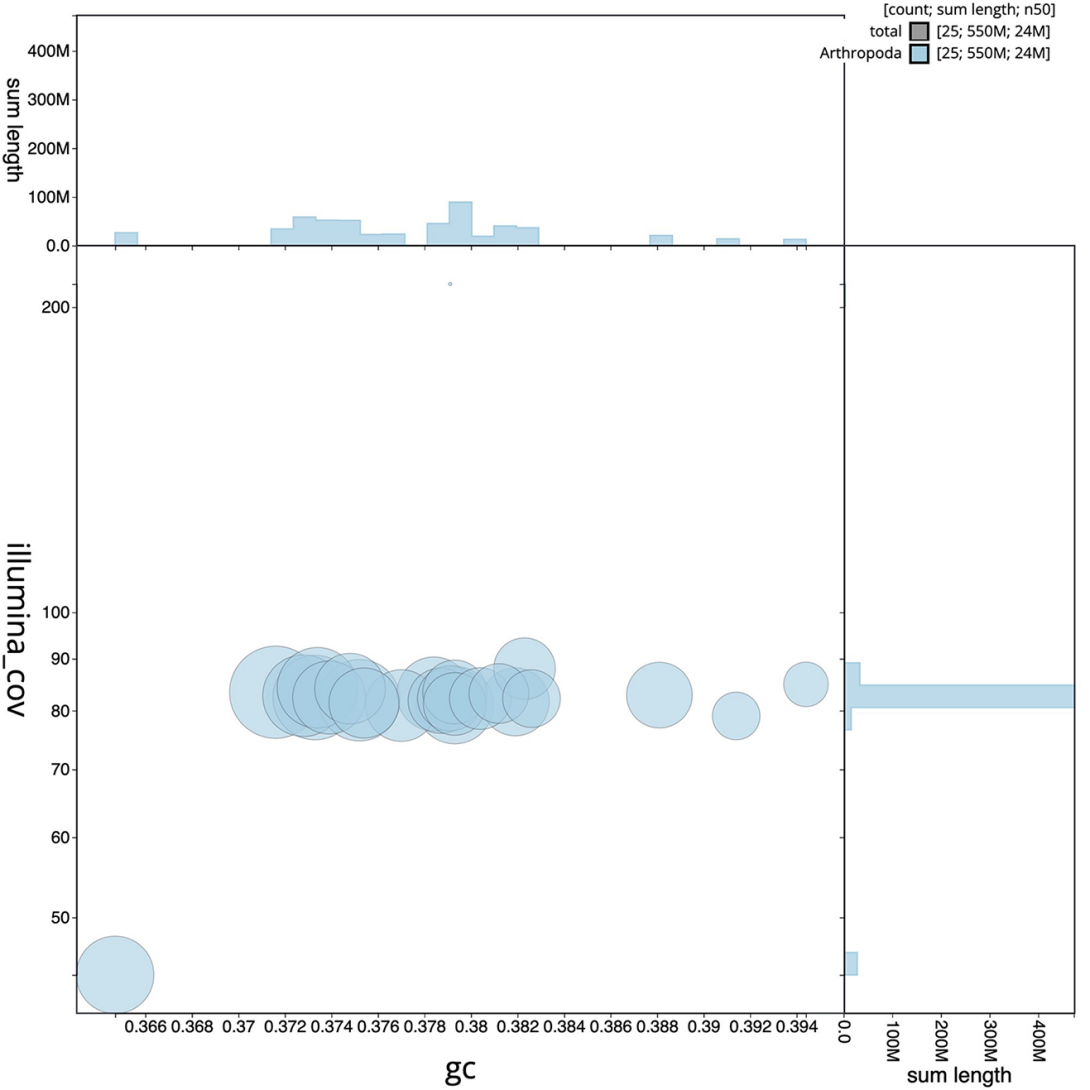
Genome assembly of *Lycaena helle*, ilHelHell1.1: BlobToolKit GC-coverage plot. Scaffolds are coloured by phylum. Circles are sized in proportion to scaffold length. Histograms show the distribution of scaffold length sum along each axis.

**Figure 4.**
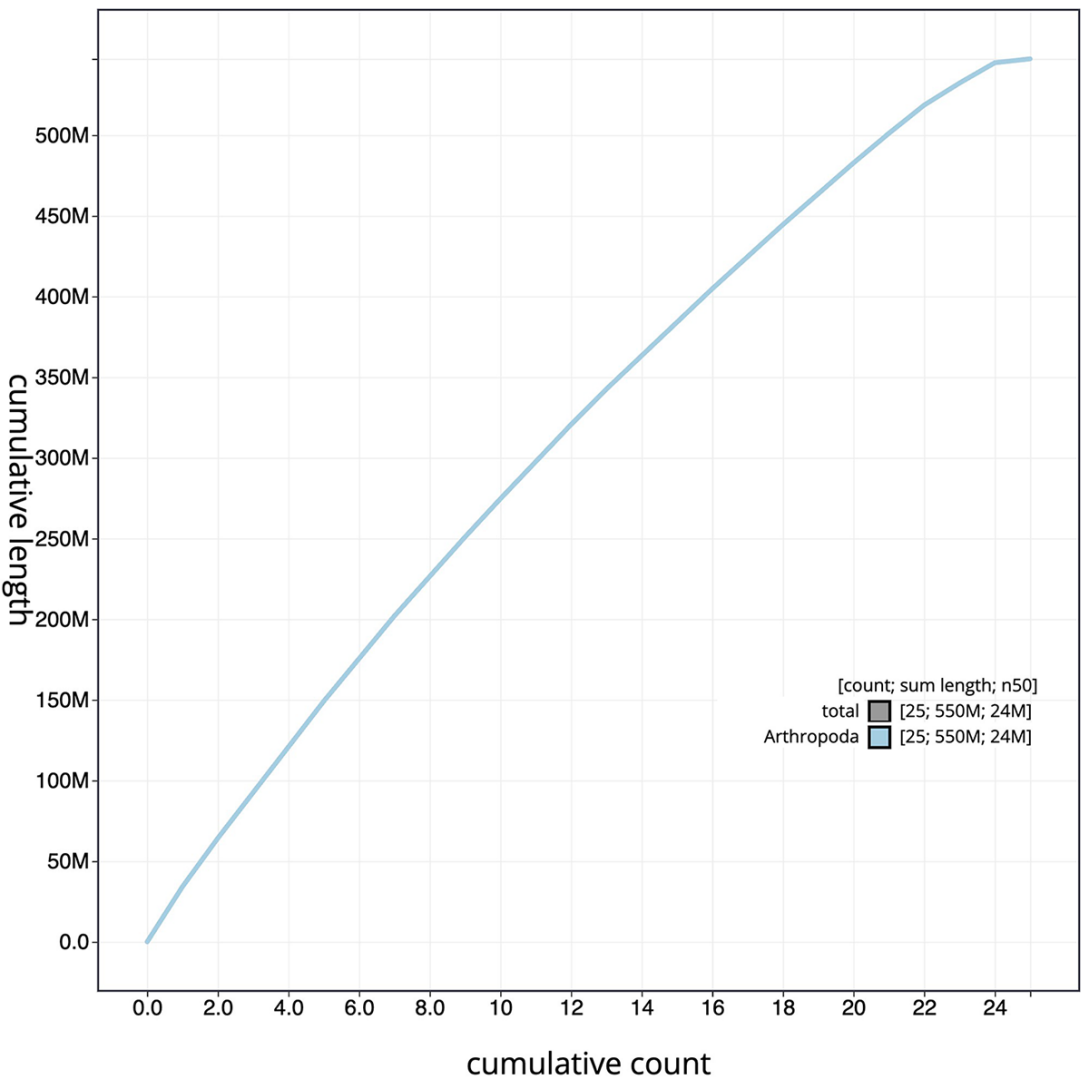
Genome assembly of of *Lycaena helle*, ilHelHell1.1: BlobToolKit cumulative sequence plot. The grey line shows cumulative length for all scaffolds. Coloured lines show cumulative lengths of scaffolds assigned to each phylum using the buscogenes taxrule.

**Figure 5.**
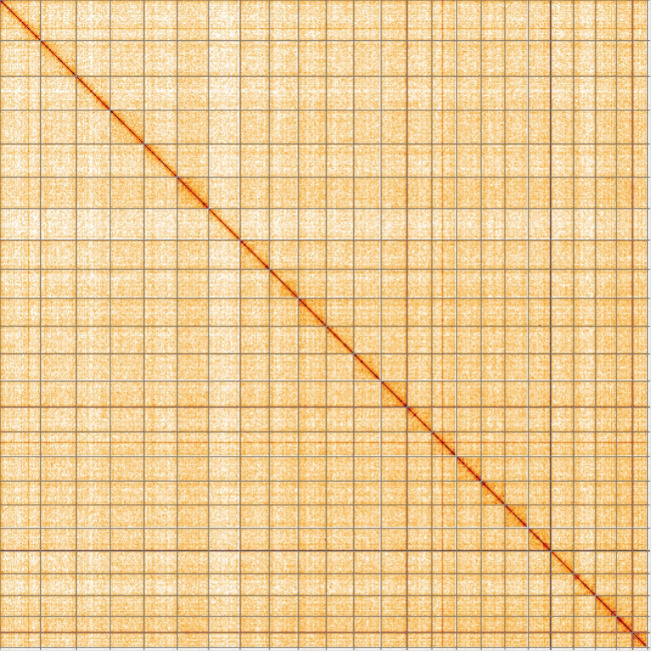
Genome assembly of *Lycaena helle*, ilHelHell1.1: Hi-C contact map of the ilHelHell1.1 assembly, visualised using HiGlass. Chromosomes are shown in order of size from left to right and top to bottom. An interactive version of this figure may be viewed at https://genome-note-higlass.tol.sanger.ac.uk/l/?d=PPZW7-xNSou542Oxv6Guzg.

The final assembly has a total length of 547.31 Mb in 25 sequence contigs with a contig N50 of 23.62 Mb ( Table 1). The snail plot in Figure 2 provides a summary of the assembly statistics, while the distribution of assembly contigs on GC proportion and coverage is shown in Figure 3. The cumulative assembly plot in Figure 4 shows curves for subsets of contigs assigned to different phyla. All (100%) of the assembly sequence was assigned to 25 chromosomal-level scaffolds, representing 23 autosomes and the W and Z sex chromosomes. Sex chromosomes were assigned based on half coverage observed in the Hi-C map Chromosome-scale scaffolds confirmed by the Hi-C data are named in order of size ( Figure 5; Table 2). While not fully phased, the assembly deposited is of one haplotype. The mitochondrial genome was also assembled and can be found as a contig within the multifasta file of the genome submission.

**Table 1.** Genome data for *Lycaena helle*, ilHelHell1.1.

**Project accession data**		
Assembly identifier	ilHelHell1.1	
Species	*Lycaena helle*	
Specimen	ilHelHell1	
NCBI taxonomy ID	2795559	
BioProject	PRJEB64648	
BioSample ID	SAMEA13959391	
Isolate information	ilHelHell1, female	
**Assembly metrics** *		*Benchmark*
Consensus quality (QV)	46.6	≥ *50*
*k*-mer completeness	94.4%	≥ *95%*
BUSCO (lepidoptera) **	C:98.4%[S:98.1%,D:0.3%],F:0.2%,M:1.4%,n:5,286	*C* ≥ *95%*
BUSCO (insecta) ***	C:99.1%[S:98.9%,D:0.2%],F:0.4%,M:0.5%,n:1,367	*C* ≥ *95%*
Percentage of assembly mapped to chromosomes	99.99%	≥ *95%*
Sex chromosomes	W and Z	*localised homologous pairs*
Organelles	Mitochondrial genome, 15.44 kb	*complete single alleles*
**Raw data accessions**		
Oxford Nanopore Technologies	ERR11775642,	
Illumina	ERR12028629 - ERR12028632	
Hi-C Illumina	ERR12028633 - ERR12028640	
PolyA Oxford Nanopore Technologies cDNAseq	ERR12028641 - ERR12028642	
**Genome assembly**		
Assembly accession	GCA_963853865.1	
Span (Mb)	547.31	
Number of contigs	25	
Contig N50 length (Mb)	23.62	
Number of scaffolds	25	
Scaffold N50 length (Mb)	23.62	
Longest scaffold (Mb)	34.21	
**Genome annotation**		
Number of protein-coding genes	20,122	
Number of non-coding genes	4,264	
Number of gene transcripts	27,022	

* Assembly metric benchmarks are adapted from column VGP-2020 of “ Table 1: Proposed standards and metrics for defining genome assembly quality” from Ref. 31.

** BUSCO scores based on the lepidoptera_odb10 BUSCO set using v5.3.2. C = complete [S = single copy, D = duplicated], F = fragmented, M = missing, n = number of orthologues in comparison. A full set of BUSCO scores is available at [interactive link].

*** BUSCO scores based on the insect_odb10 BUSCO set using v5.3.2.

**Table 2.** Chromosomal pseudomolecules in the genome assembly of *Lycaena helle*, ilHelHell1.

Chromosome	INSDC accession	Length (Mb)	GC_Percent
**1**	OY971414.1	34.21	37
**2**	OY971415.1	30.02	37.5
**3**	OY971416.1	28.47	37.5
**4**	OY971417.1	28.44	37.5
**5**	OY971418.1	27.98	37.5
**6**	OY971419.1	26.45	38
**7**	OY971421.1	24.71	38
**8**	OY971422.1	24.26	37.5
**9**	OY971423.1	23.62	37.5
**10**	OY971424.1	23.15	37.5
**11**	OY971425.1	22.85	37.5
**12**	OY971426.1	21.95	38
**13**	OY971427.1	20.89	39
**14**	OY971428.1	20.75	38
**15**	OY971429.1	20.65	38
**16**	OY971430.1	20.11	38
**17**	OY971431.1	19.62	38
**18**	OY971432.1	19.14	38
**19**	OY971433.1	19.11	38
**20**	OY971434.1	18.34	38
**21**	OY971435.1	17.49	38.5
**22**	OY971436.1	13.66	39
**23**	OY971437.1	12.6	39.5
**W**	OY971438.1	2.41	38
**Z**	OY971420.1	26.43	36.5
**Mitochondrion**	OY971439.1	0.02	17.5

**Figure 6.**
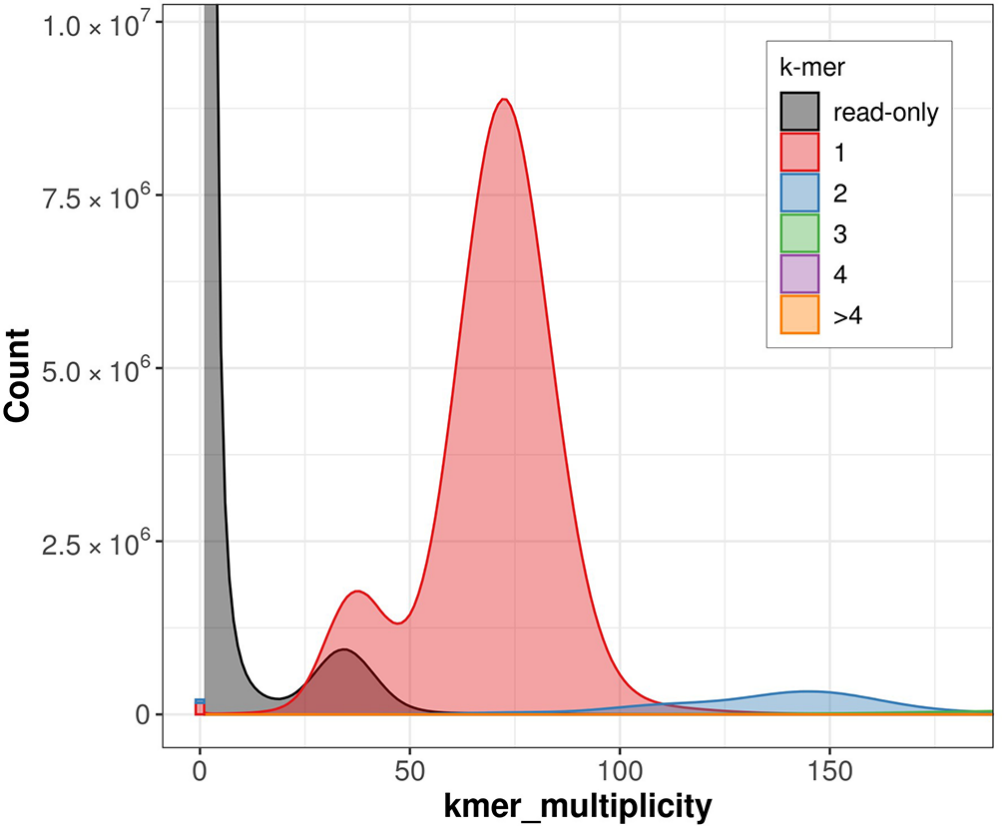
Merqury assembly spectrum plot. This plot tracks the multiplicity of each *k*-mer found in the Illumina read set and colors it by the number of times it is found in the assembly.

The estimated Quality Value (QV) of the final assembly is 46.6 with *k*-mer completeness of 94.4%. The k-mer spectra profile generated by Merqury ( Figure 6) indicates high completeness, low error and low artificial duplication rates. The assembly has a BUSCO v5.3.2 completeness of 98.4% (single = 98.1%, duplicated = 0.3%), using the lepidoptera_odb10 reference set ( *n* = 5,286).

**Table 3.** Software tools: versions and sources.

Software tool	Version	Source
BlobToolKit	4.1.7	https://github.com/blobtoolkit/blobtoolkit
BUSCO	5.3.2	https://gitlab.com/ezlab/busco
BWA	V0.7.17-r1188	https://bio-bwa.sourceforge.net/bwa.shtml
Flye	2.9.1 -b1780	https://github.com/fenderglass/Flye
Minimap2	2.14	https://github.com/lh3/minimap2
Minimap2	2.24-r1122	https://github.com/lh3/minimap2
Mitos	2.1.3	http://mitos.bioinf.uni-leipzig.de/
Merqury	1.3	https://github.com/marbl/merqury
NextDenovo	2.4.0	https://github.com/Nextomics/NextDenovo
PretextView	0.2.5	https://github.com/wtsi-hpag/PretextView
purge_dups	1.2.5	https://github.com/dfguan/purge_dups
Pairtools	0.3.0	https://github.com/open2c/pairtools
sanger-tol/genomenote	v1.0	https://github.com/sanger-tol/genomenote
sanger-tol/readmapping	1.1.0	https://github.com/sanger-tol/readmapping/tree/1.1.0
Samtools	1.9	http://www.htslib.org/

### Genome annotation

The *Lycaena helle* genome assembly (GCA_963853865.1) was annotated using the CNAG structural genome annotation pipeline ( Table 1; https://github.com/cnag-aat/Annotation_AAT). The resulting annotation includes 22,758 transcribed mRNAs from 20,122 protein-coding and 4,264 non-coding genes. There are 1.13 coding transcripts per gene and 6.1 exons per transcript.

#### Ethical considerations

All steps required for proper transportation of the material were followed. The collection and transport of the samples had the authorization of the Andorran government. The capture was the resolution number 312745, Andorra la Vella, 19/05/2021. The exportation authorization from Andorra to Spain had the expedient number of 21AD0616/02P valid until the 16/12/2021 with the register number of 9326594 and with date of authorization 16/06/2021. The import was also authorized by the Spanish customs. At the time the samples were sent from Andorra to Spain, the species was not included in the CITES list.

## Data Availability

The data for this study have been deposited in the European Nucleotide Archive (ENA) at EMBL-EBI under accession number PRJEB64648 ( https://identifiers.org/ena.embl/PRJEB64648). ^51^ This project provides the sequencing data, genome assembly and annotation of *Lycaena helle*, the violet copper butterfly. The data was produced in collaboration with the ERGA sample ambassador Vanessa Arroyo (Andorra), the ERGA Pilot Project team and the Catalan Initiative for the Earth Biogenome Project ( https://www.biogenoma.cat/). The European Reference Genome Atlas ( https://www.erga-biodiversity.eu/) and the Catalan Initiative for the Earth Biogenome Project are affiliated projects under the umbrella of the Earth BioGenome Project. Please see the ERGA Pilot Project Data Sharing and Management Policy ( https://doi.org/10.5281/ZENODO.8091290) prior to publication using this data. Figshare: Data sets used and Supplementary Table 1. http://www.doi.org/10.6084/m9.figshare.12185193. ^52^
