# Supplementary material for: The genome sequence of the Violet Copper, Lycaena helle (Denis & Schiffermüller, 1775)
Source: F1000Res. 2025 Jan 10;14:60. [Version 1] doi: 10.12688/f1000research.156485.1 (PMC12174908; doi:10.12688/f1000research.156485.1)
Supplement: Peer review history

# Reviewer report: Reviewer response for version 1

**DOI:** 10.5256/f1000research.171804.r382332

**Published:** 2025-06-17

**Authors:** Sivasankaran Kuppusamy

**Affiliations:** 1 Loyola College, Chennai, Tamil Nadu, India

The chromosome level genome of Violet Copper, *Lycaena helle* (Denis & Schiffermuller, 1775) was sequenced and assembled. Authors have used appropriate DNA isolation and sequencing techniques. Through the sequence assembly authors have obtained 547.31 Mb and detected 25 chromosomal pseudomolecules including sex chromosomes. They also identified protein-coding genes, non-coding genes and gene transcripts.

**Minor comments on the manuscripts**

 The first sentence of the Background can be written as “The genome of the Violet Copper, *Lycaena helle* (Denis & Schiffermüller, 1775)………………….

 The sentence “The adult butterfly feeds on the nectar of different plant species” can be deleted.

 Authors have used the full form of Genus name *Lycaena* throughout the text. The genus name can be used in a short form like *L. helle*.

 Subheading Genome Annotation third paragraph penultimate sentence “rounds of PASA annotation *updates*. Update is in italics. Authors can change italics to normal letters

 Above all, I confirm that the manuscript meets the necessary scientific standard and is suitable for indexing"

Are the datasets clearly presented in a usable and accessible format, and the assembly and annotation available in an appropriate subject-specific repository?

Yes

Are sufficient details of the sequencing and extraction, software used, and materials provided to allow replication by others?

Yes

Are the rationale for sequencing the genome and the species significance clearly described?

Yes

Are the protocols appropriate and is the work technically sound?

Yes

Reviewer Expertise:

Phylogenetic analysis of Noctuoidea moths using mitochondrial genome

I confirm that I have read this submission and believe that I have an appropriate level of expertise to confirm that it is of an acceptable scientific standard.

# Reviewer report: Reviewer response for version 1

**DOI:** 10.5256/f1000research.171804.r382340

**Published:** 2025-05-26

**Authors:** Panagiotis Ioannidis

**Affiliations:** 1 Foundation for Research & Technology - Hellas, Crete, Greece

This paper by Pladevall and colleagues reports the genome sequencing, assembly, and annotation of the lepidopteran Lycaena helle.

 I noticed that even though the authors ran a BUSCO evaluation for the genome assembly (which gave completeness scores >98% with the lepidopteran set), they didn't run BUSCO in the predicted gene set, as well. Running BUSCO on the gene set is of paramount importance because it gives a picture of how good the gene set is, compared to the assembly. More specifically and since the complete lepidopteran BUSCOs are 98.4% for the genome, the same score for the gene set should ideally be at least 98.4%. If it's <98.4%, then something has gone wrong with gene prediction. The bigger the difference, the more substantial the problem. And if the difference is too large, then gene prediction should be repeated after changing, for example, the set of transcript and/or protein evidence.

 In summary, it would be nice if the authors provided the BUSCO scores for the predicted gene set as well. I understand, of course, that repeating gene prediction (if such a thing is needed) can't be done now. However, it will be useful for anyone who wants to use this gene set, as it will show how good it is compared to the assembly.

Are the datasets clearly presented in a usable and accessible format, and the assembly and annotation available in an appropriate subject-specific repository?

Yes

Are sufficient details of the sequencing and extraction, software used, and materials provided to allow replication by others?

Yes

Are the rationale for sequencing the genome and the species significance clearly described?

Yes

Are the protocols appropriate and is the work technically sound?

Yes

Reviewer Expertise:

Insect genomics, bioinformatics

I confirm that I have read this submission and believe that I have an appropriate level of expertise to confirm that it is of an acceptable scientific standard.
